# Recent Progress in Crystalline Borates with Edge-Sharing BO_4_ Tetrahedra

**DOI:** 10.3390/molecules28135068

**Published:** 2023-06-28

**Authors:** Jing-Jing Li, Wei-Feng Chen, You-Zhao Lan, Jian-Wen Cheng

**Affiliations:** Key Laboratory of the Ministry of Education for Advanced Catalysis Materials, Institute of Physical Chemistry, Zhejiang Normal University, Jinhua 321004, China

**Keywords:** borate, edge-sharing, fundamental building blocks, structural chemistry

## Abstract

Crystalline borates have received great attention due to their various structures and wide applications. For a long time, the corner-sharing B–O unit is considered a basic rule in borate structural chemistry. The Dy_4_B_6_O_15_ synthesized under high-pressure is the first oxoborate with edge-sharing [BO_4_] tetrahedra, while the KZnB_3_O_6_ is the first ambient pressure borate with the edge-sharing [BO_4_] tetrahedra. The edge-sharing connection modes greatly enrich the structural chemistry of borates and are expected to expand new applications in the future. In this review, we summarize the recent progress in crystalline borates with edge-sharing [BO_4_] tetrahedra. We discuss the synthesis, fundamental building blocks, structural features, and possible applications of these edge-sharing borates. Finally, we also discuss the future perspectives in this field.

## 1. Introduction

Borates show rich structural chemistry and have broad applications as birefringent materials and nonlinear optical (NLO) materials [1,2,3,4,5,6,7,8,9,10,11,12,13,14,15,16,17,18,19,20,21,22,23,24,25,26,27,28,29,30,31]. The famous KBe_2_BO_3_F_2_ (KBBF), LiB_3_O_5_ (LBO), and *β*-BaB_2_O_4_ (*β*-BBO) crystals are used to generate ultraviolet (UV) or deep-UV lasers through cascaded frequency conversion in practical application [32,33,34]. *α*-BaB_2_O_4_ (*α*-BBO) is an excellent UV birefringent crystal with a wide transparency window from 190 nm to 3500 nm and a large birefringence of 0.15 at 266 nm [35]. To date, the number of synthetic borates and borate minerals are over 3900 in the documented literature [1]. Three types of B–O units of linear [BO_2_], triangular [BO_3_], and tetrahedral [BO_4_] are observed in these borates in which linear [BO_2_] with *sp* hybridized chemical bonds are extremely rare; only 0.1% of borates contain the linear [BO_2_] configuration. M_5_Ba_2_(B_10_O_17_)_2_(BO_2_) (M = K, Rb) and NaRb_6_(B_4_O_5_(OH)_4_)_3_(BO_2_) are three typical examples; the former two compounds contain unusual [BO_2_] with the traditional [BO_3_] and [BO_4_] units and exhibit suitable birefringence (Δ*n* = 0.06) and transparency windows down to the deep-UV region (<190 nm) [36,37]. Theoretical analyses reveal that the [BO_3_] and [BO_4_] units have the smaller polarizability anisotropy compared with linear [BO_2_]. While the latter one is the first noncentrosymmetric and chiral structure with the linear [BO_2_] unit and displays a weak second-harmonic generation response (SHG) (0.1 × SiO_2_) and wide transparency of about 21.2% at 200 nm [38].

In 2021, Pan and coworkers summarized the synthesis, fundamental building blocks (FBBs), symmetries, structure features, and functional properties of the reported anhydrous borates [1]. The FBBs of polynuclear borates are generally formed by corner-/edge-sharing [BO_3_] and [BO_4_] units. Cs_3_B_7_O_12_ contains a large FBB with 63 boron atoms in which 35 (or 37) BO_3_ triangles and 28 (or 26) BO_4_ tetrahedra are linked to form thick anionic sheets stacked along the *c* direction [39]. Mg_7_@[B_69_O_108_(OH)_18_] contains 42 [BO_3_] triangles and 27 [BO_4_] tetrahedra; it exhibits a supramolecular framework with hexagonal snowflake-like channels; unique triple-helical ribbons are found in {B_69_} FBBs [40]. This huge [B_69_O_108_(OH)_18_] cluster represents the largest FBB in borates. The FBBs can further polymerize into 1D chains, 2D layers, and 3D networks [41,42,43,44,45,46,47,48,49]. For example, we obtained three alkali and alkaline earth-metal borates, namely Ba_2_B_10_O_16_(OH)_2_·(H_3_BO_3_)(H_2_O), Na_2_B_10_O_17_·H_2_en, and Ca_2_[B_5_O_9_]·(OH)·H_2_O [41,42,43], in which pentaborates are used to construct a single-layered structure, 2D microporous layers, and a 3D network, respectively. Ca_2_[B_5_O_9_]·(OH)·H_2_O is impressive with a dense net consisting of *pcu* B−O net and *dia* Ca−O net and exhibits a short UV cutoff edge below 200 nm and a strong SHG response of ~three times that of KH_2_PO_4_ (KDP) [43].

In 2002, Huppertz and coworkers reported the high-pressure synthesis of Dy_4_B_6_O_15;_ it is the first oxoborate with an edge-sharing BO_4_ tetrahedra [50]. The edge-sharing [BO_4_] tetrahedra in Dy_4_B_6_O_15_ changes the rule of corner-sharing [BO_3_]/[BO_4_] units in borate structural chemistry. In addition, it is considered that the extreme synthetic conditions, such as high pressure, is necessary for edge-sharing borates. In 2010, the discovery of KZnB_3_O_6_ changed this view; KZnB_3_O_6_ represents the first ambient pressure edge-sharing [BO_4_]-containing borate [51]. To date, edge-sharing [BO_4_]-containing borates are still rare; less than 1% of borates contain edge-sharing BO_4_ tetrahedra. Over the past decade, the synthesis, crystal structures, and properties of KBBF-like borates [52], fluorooxoborates [53,54], high-temperature borates [55], high-pressure borates [56], *f*-element borates [57], zincoborates [58,59], aluminoborates [60,61], borogermanates [62], hybrid *d*- or *p*-block metal borates [63], and hydrated borates with non-metal or transition-metal complex cations have been well reviewed [64]. Herein, we give a detailed summary of the recent progress in crystalline borates with edge-sharing BO_4_ tetrahedra. These edge-sharing borates can be grouped into two types in terms of their synthetic method: (i) high pressure synthesis of borates with edge-sharing [BO_4_] tetrahedra and (ii) ambient pressure synthesis of borates with edge-sharing [BO_4_] tetrahedra. We discuss the synthesis, FBBs, structural features, potential applications, and future perspectives of edge-sharing borates.

## 2. High Pressure Synthesis of Borates with Edge-Sharing [BO_4_] Tetrahedra

The existence of uncommon edge-sharing [BO_4_] tetrahedra disobeys Pauling’s third rule. The borates containing the so-called edge-sharing [B_2_O_6_] dimer were initially believed to be obtained only under extreme conditions, such as high temperature and high pressure. Since the first case of this species was discovered, multi-anvil high-pressure synthesis is the dominant route to obtain the new edge-sharing [BO_4_] tetrahedra-containing borates. Up to now, there are 26 high-pressure edge-sharing borates within the scope of discussion. Boron atoms tend to coordinate with four O atoms to form [BO_4_] tetrahedra under a high-pressure environment, as evidenced by most of these high-pressure compounds constructed merely from [BO_4_] tetrahedra. Even in [BO_3_]-containing borates, such as high-pressure AB_3_O_5_, the proportion of the [BO_3_] triangle is only 1/3.

### 2.1. Rare Earth Borates

**Figure 1:**
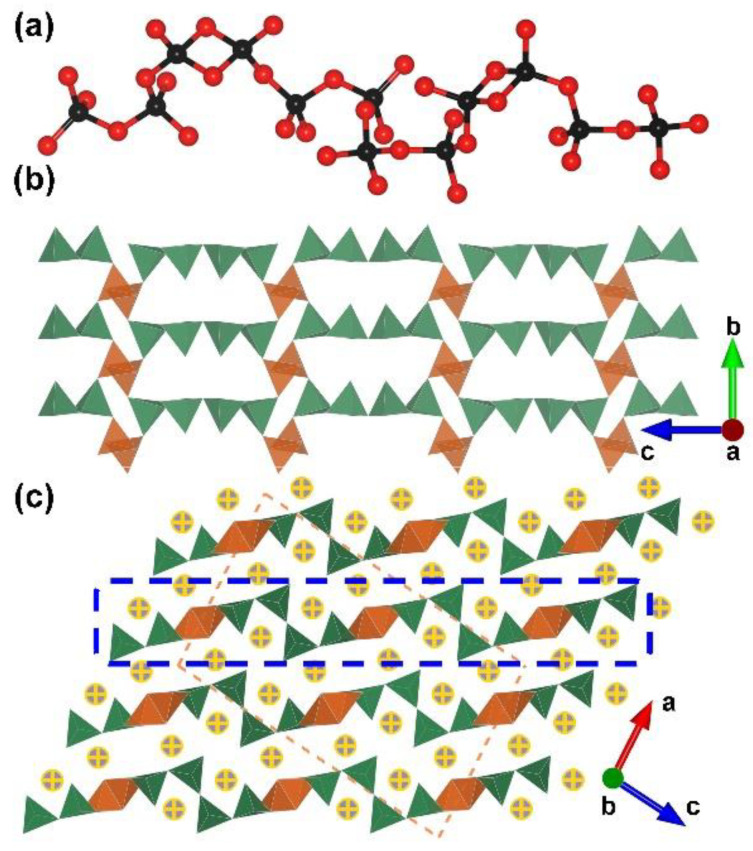
(**a**) The [B_12_O_35_] FBB; (**b**) the ^2^[B_6_O_15_]_∞_ corrugated layer; (**c**) the total structure of RE_4_B_6_O_15_ (RE = Dy and Ho) along [010] direction. Key: cross-centered purple ball, rare earth atom; black ball, B atom; red ball, O atom; orange/olive tetrahedron, edge/vertex-sharing [BO_4_]; purple triangle, [BO_3_].

RE_4_B_6_O_15_ (RE = Dy and Ho). Dy_4_B_6_O_15_ is the first reported metal borate with edge-sharing [BO_4_] tetrahedra; it was obtained under high-temperature (1273 K) and high-pressure (8 Gpa) conditions by Huppertz et al. in 2002 [50]. Shortly after, isostructural Ho-analogues was prepared under the same extreme high-pressure condition in 2003 [65]. The RE_4_B_6_O_15_ series crystallize in the monoclinic crystal system with the space group of *C*2/*c* (no. 15); their structures exhibit corrugated ^2^[B_6_O_15_]_∞_ layers formed by the linkage of the adjacent [B_12_O_35_] clusters (Figure 1b). The large [B_12_O_35_] cluster, incorporating edge-sharing and corner-sharing [BO_4_] tetrahedra with the ratio of 8:4, can be considered as the FBB of RE_4_B_6_O_15_ (Figure 1a). Furthermore, the interlayer rare earth ions connect these corrugated layers to form the final 3D structures (Figure 1c). The multi-anvil techniques, which can offer external pressures, accelerate the discovery of borates with unusual edge-sharing [BO_4_] tetrahedra and initiate the era of exploring such borates under multi-anvil high-pressure conditions.

**Figure 2:**
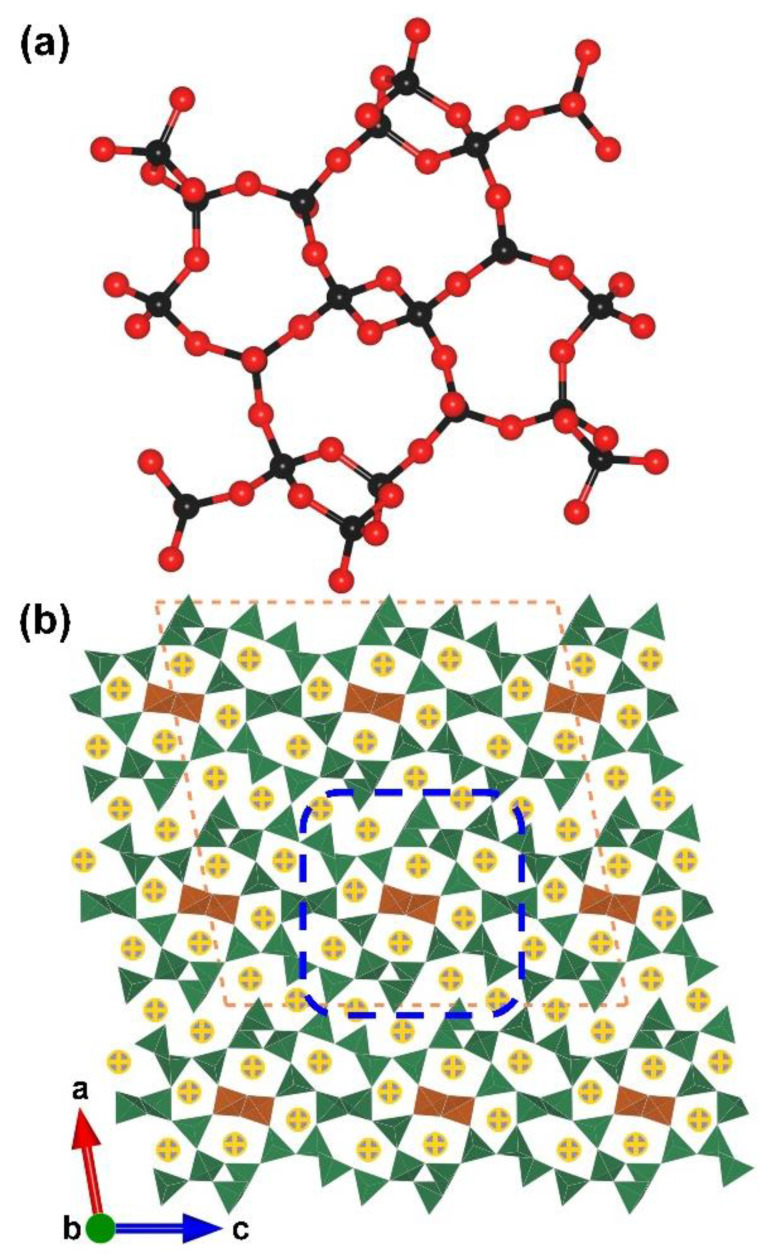
(**a**) The [B_20_O_55_] FBB; (**b**) the total structure of RE_2_B_4_O_9_ (RE = Sm, Eu, Gd, Tb, Dy, Ho, and Y) along [010] direction. Key: cross-centered purple ball, rare earth atom; black ball, B atom; red ball, O atom; orange/olive tetrahedron, edge/vertex-sharing [BO_4_].

α-RE_2_B_4_O_9_ (RE = Sm, Eu, Gd, Tb, Dy, Ho and Y). *α*-RE_2_B_4_O_9_ borates (RE = Sm, Eu, Gd, Tb, Dy, Ho and Y) are another rare earth borate series with edge-sharing [BO_4_] tetrahedra reported in the period of 2002 to 2017 [66,67,68,69]. Similar to the RE_4_B_6_O_15_ series, the *α-*RE_2_B_4_O_9_ series crystallize in the same space group (*C*2/*c*, no. 15) in which all the incorporating boron atoms are four-coordinated. In these structures, the complex [B_20_O_55_] FBB is comprised with edge- and corner-sharing [BO_4_] tetrahedra according to the ratio of 18:2 (Figure 2a and blue blanket in Figure 2b). With respect to the whole covalent B–O framework of RE_4_B_6_O_15_, the ^3^[B_6_O_15_]_∞_ network is formed by the linkage of [B_20_O_55_] FBBs, the rare earth cations located in the channels (Figure 2b).

**Figure 3:**
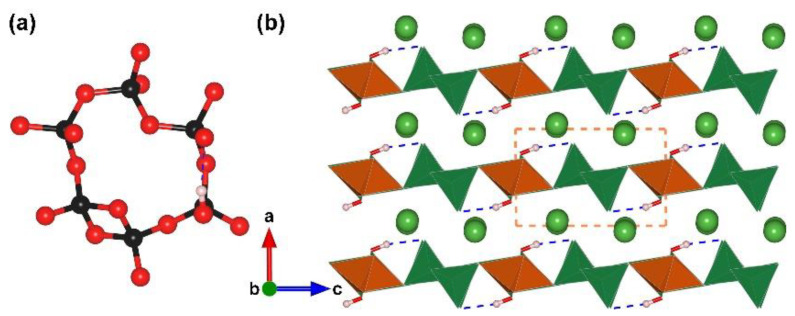
(**a**) The [B_6_O_16_(OH)] FBB of La_3_B_6_O_13_(OH); (**b**) the total structure of La_3_B_6_O_13_(OH) along [010] direction. Key: green ball, La atom; black ball, B atom; red ball, O atom; small pink ball, H atom; orange/olive tetrahedron, edge/vertex-sharing [BO_4_].

La_3_B_6_O_13_(OH). During the synthetic process, the replacement of the anhydrous boron source with boric acid, hydrated borates, or borates containing water molecules are sometimes obtained. La_3_B_6_O_13_(OH) is the first SHG-active edge-sharing [BO_4_] tetrahedra-containing borate [70]. This compound was obtained by a high-pressure/high-temperature condition at 6 GPa and 1673 K and was immediately identified as an NLO crystal by Huppertz et al. in 2020. It crystallizes in the chiral space group, *P*2_1_ (no. 4), and presents a 2D ^2^[B_6_O_13_(OH)]_∞_ layered structure with La ions located between the layers (Figure 3). The FBB of La_3_B_6_O_13_(OH) features a ‘sechser’-ring, which is constructed of one [B_2_O_6_], three vertex-sharing [BO_4_], and one [BO_3_(OH)] (Figure 3a). The [B_6_O_16_(OH)] FBBs are linked into a 2D ^2^[B_6_O_13_(OH)]_∞_ layer along the *bc* plane, which further stack along [100] direction with La ions residing in the interlayer space (Figure 3b). Although La_3_B_6_O_13_(OH) crystallizes in a noncentrosymmetric space group, its basic B–O units in the lattice are all the non-*π*-conjugated tetrahedral. La_3_B_6_O_13_(OH) displays a relatively weak SHG effect. Compared to the non-*π*-conjugated [BO_4_] tetrahedron with negligible hyperpolarization, the *π*-conjugated motifs represented by planar [BO_3_] and [B_3_O_6_] in the borate system are superior NLO-active functional modules, and thus, the powder SHG response of La_3_B_6_O_13_(OH) based on the Kurtz–Perry method is as weak as 2/3 times that of quartz.

### 2.2. Transition Metal Borates

**Figure 4:**
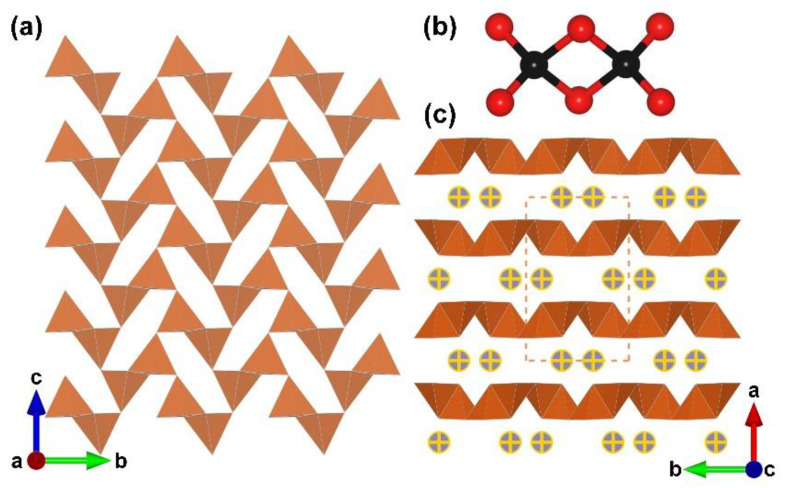
(**a**) The ^2^[B_2_O_4_]_∞_ layer expanding in the *bc* plane; (**b**) the [B_2_O_6_] FBB; (**c**) the total structure of TMB_2_O_4_ (TM = Ni, Fe and Co) along [001] direction. Key: cross-centered purple ball, divalent transition metal atom; black ball, B atom; red ball, O atom; orange tetrahedron, edge-sharing [BO_4_].

TMB_2_O_4_ (TM = Ni, Fe and Co). Previous research on edge-sharing [BO_4_]-containing borates mainly focus on lanthanide borates. Later, researchers achieved the combination of transition metal and edge-sharing [BO_4_] tetrahedra. From 2007 to 2010, a series of high-pressure transition metal borates, TMB_2_O_4_ (TM = Ni, Fe and Co), were discovered by Huppertz and coworkers [71,72,73]. All boron atoms in this species are four-coordinated, and the FBB is the simplest [B_2_O_6_] cluster (Figure 4b). Each edge-shared [B_2_O_6_] dimmer is linked to four surrounding [B_2_O_6_] units through *μ*_2_-O atoms, resulting in a dense 2D ^2^[B_2_O_4_]_∞_ layer with six-member rings (6 MRs) (Figure 4a). The stacking of ^2^[B_2_O_4_]_∞_ layer along [100] direction is further linked by interlayer TM ions, which leads to the final structure of TMB_2_O_4_ (Figure 4c).

**Figure 5:**
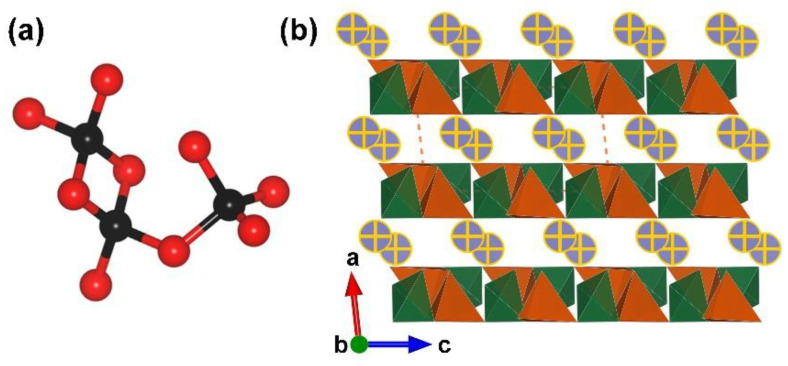
(**a**) The [B_3_O_9_] FBB; (**b**) the total structure of HfB_2_O_5_ along [010] direction. Key: cross-centered purple ball, Hf atom; black ball, B atom; red ball, O atom; orange tetrahedron, edge-sharing [BO_4_].

γ-HfB_2_O_5_. In 2021, the *γ*-phase of HfB_2_O_5_, which incorporates edge-sharing [BO_4_] tetrahedra, was obtained under extreme pressure (120 GPa) by Huppertz [74]. *γ*-HfB_2_O_5_ crystallizes in the centrosymmetric monoclinic space group, *P*2_1_/*c* (no. 14). The tetravalent transition metal Hf^4+^ cation displays higher coordination numbers than divalent cations, and the FBB in *γ-*HfB_2_O_5_ is changed to [B_3_O_9_] with the additional one vertex-sharing [BO_4_] (Figure 5a). Similar to the stuctures of TMB_2_O_4_ series, the structure of γ-HfB_2_O_5_ borate also shows layered sheets with Hf ions filling the interlayer space (Figure 5b). It is interesting to note that *β*-HfB_2_O_5_ was synthesized at 7.5 GPa in the multi-anvil press, upon further compression up to 120 GPa, a shrinkage of the cell parameters during the compression process was observed, and finally the *β*-phase is transformed to the *γ-*phase. The layer in *β*-HfB_2_O_5_ contains four MRs and eight MRs by the corner-sharing BO_4_ tetrahedra, while *γ*-HfB_2_O_5_ contains ten MRs, including the edge-sharing BO_4_ tetrahedra. Edge-sharing BO_4_ tetrahedra in new phase *γ*-HfB_2_O_5_ shows exceptionally short B–O and B⋯B distances. The coordination number of the Hf^4+^ cations in *γ-*HfB_2_O_5_ increased to nine in comparison to eight in its ambient pressure counterpart.

**Figure 6:**
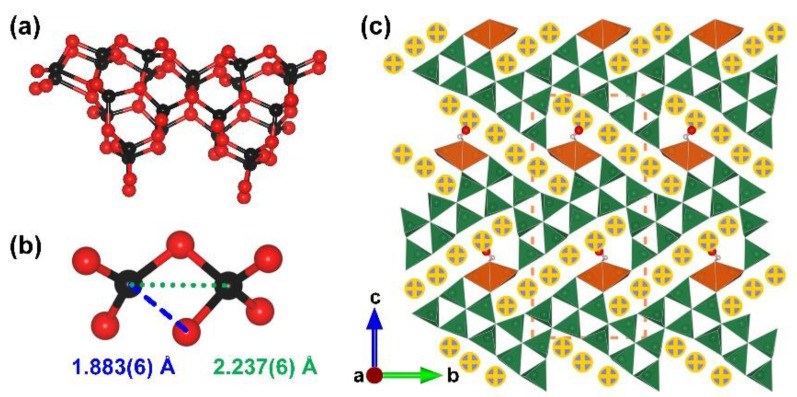
(**a**) The [B_24_O_54_] FBB of M_6_B_22_O_39_·H_2_O (M = Fe and Co); (**b**) coordination spheres of boron atoms B(11) and B(8) in Fe_6_B_22_O_39_·H_2_O; (**c**) the total structure of M_6_B_22_O_39_·H_2_O (M = Fe and Co) along [100] direction. Key: cross-centered purple ball, Fe/Co atom; black ball, B atom; red ball, O atom; small pink ball, H atom; orange/olive tetrahedron, edge/vertex-sharing [BO_4_].

M_6_B_22_O_39_·H_2_O (M = Fe and Co). The first two acentric edge-sharing [BO_4_] tetrahedra-containing borates M_6_B_22_O_39_·H_2_O (M = Fe and Co) were prepared under the high-pressure (6 GPa) and high-temperature (880 °C for Fe and 950 °C for Co) conditions in a Walker-type multi-anvil apparatus by Huppertz et al. in 2010 [75]. The M_6_B_22_O_39_·H_2_O series crystallize in a noncentrosymmetric orthorhombic space group, *Pmn*2_1_ (no. 31). The unusually long B–O bond lengths as well as the short distances between the two boron cores are shown in the structure, which indicates the successful capture of intermediate states on the way to edge-sharing [BO_4_] tetrahedra. The structure of M_6_B_22_O_39_·H_2_O shows a 3D [B_22_O_39_]_∞_ anhydrous B–O framework with the Fe or Co ions and water molecules located in the structural channels (Figure 6a,c). Specifically, taking Fe_6_B_22_O_39_·H_2_O as an example, the B(11), O(2), O(15), and O(24) in the structure are not strictly in the same plane, and the B(11)-O(16) bond length (1.883(6) Å) is obviously longer than the common B–O distances (Figure 6b). The group of B(11) center and its three coordinated O(2,15,24) atoms as well as the neighboring O(16) can be regarded as a highly twisted polyhedron or the intermediate states between [BO_3_] tringle and [BO_4_] tetrahedron. The discovery of M_6_B_22_O_39_·H_2_O is helpful for understanding the dynamic process from the vertex-sharing [BO_3_] + [BO_4_] model to edge-sharing [BO_4_] + [BO_4_] model.

**Figure 7:**
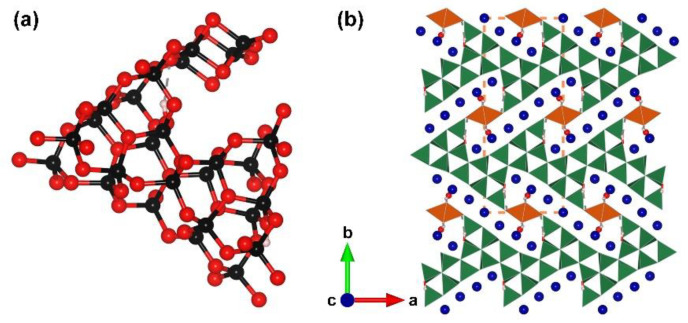
(**a**) The [B_24_O_48_(OH)_2_] FBB of Co_7_B_24_O_42_(OH)_2_·2H_2_O; (**b**) the total structure of Co_7_B_24_O_42_(OH)_2_·2H_2_O along [001] direction. Key: navy ball, Co atom; black ball, B atom; red ball, O atom; small pink ball, H atom; orange/olive tetrahedron, edge/vertex-sharing [BO_4_].

Co_7_B_24_O_42_(OH)_2_·2H_2_O. Although the cobalt hydrated borate Co_7_B_24_O_42_(OH)_2_·2H_2_O crystallizes in a centrosymmetric space group, *Pbam* (no. 55), it shares similar structural characteristics with Co_6_B_22_O_39_·H_2_O. This species was prepared under high-pressure (6 GPa) and high-temperature (1153 K) conditions by Huppertz et al. in 2012 [76]. The complex FBB of Co_7_B_24_O_42_(OH)_2_·2H_2_O is comprised of twenty-two corner- and two edge-sharing [BO_4_] tetrahedra with two hydroxy group locating in the mirror plane (Figure 7a). The structure of Co_7_B_24_O_42_(OH)_2_·2H_2_O shows the ^3^[B_24_O_42_(OH)_2_]_∞_ framework with Co ions and water molecules located in the structural channels (Figure 7b).

### 2.3. Borates with Monovalent or Divalent A-Site Cations

**Figure 8:**
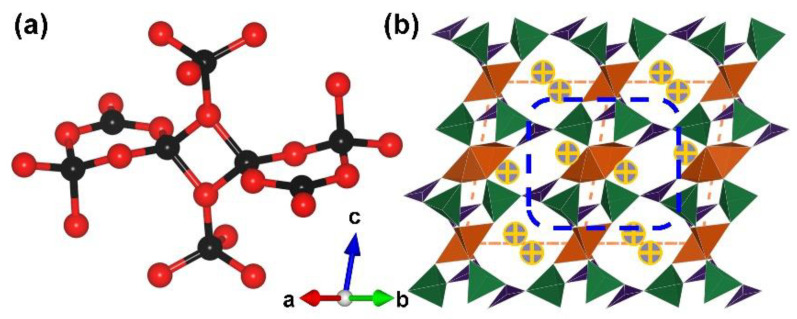
(**a**) The [B_8_O_20_] FBB; (**b**) the total structure of AB_3_O_5_ along [110] direction. Key: cross-centered purple ball, monovalent cation; black ball, B atom; red ball, O atom; orange/olive tetrahedron, edge/vertex-sharing [BO_4_]; purple triangle [BO_3_].

AB_3_O_5_ [A = K, NH_4_, Rb, Tl and Cs_1-x_(H_3_O)_x_ (x = 0.5–0.7)]. During the period of 2011 to 2014, the AB_3_O_5_ series [A = K, NH_4_, Rb, Tl and Cs_1-*x*_(H_3_O)*_x_* (*x* = 0.5–0.7)] were synthesized under high-pressure/high-temperature conditions by Huppertz et al. [77,78,79,80]. KB_3_O_5_ is the first compound with various configurations, including corner-sharing [BO_3_], corner-sharing [BO_4_], and edge-sharing [BO_4_]. The FBB of the isostructural AB_3_O_5_ contains two [BO_3_] triangles, four corner-sharing [BO_4_] tetrahedra, and two edge-sharing [BO_4_] tetrahedra (Figure 8a). It should be noted that the [B_2_O_6_] rings in AB_3_O_5_ can be regarded as six connected nodes; the two endocyclic O atoms of each [B_2_O_6_] ring are further connected with two corner-sharing [BO_4_] tetrahedra. The total structures of the AB_3_O_5_ series exhibit 3D B–O anionic skeletons with monovalent cations locating the structural channels (Figure 8b). Although the boron source in the synthesis of AB_3_O_5_ series are boric acid, only the Cs_1-*x*_(H_3_O)*_x_*B_3_O_5_ (*x* = 0.5–0.7) phase successfully incorporates oxonium ions into its structure.

**Figure 9:**
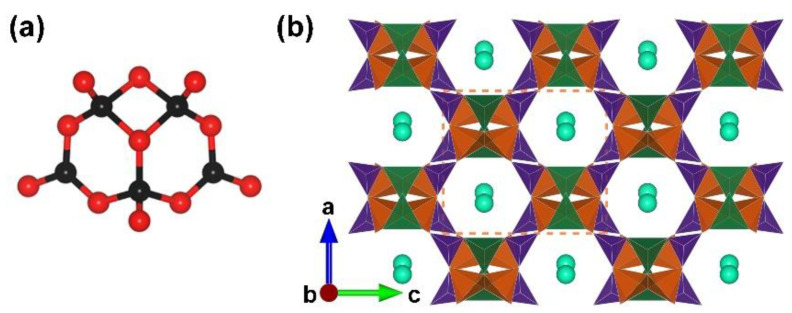
(**a**) The [B_5_O_11_] FBB; (**b**) the total structure of CsB_5_O_8_ along [010] direction. Key: cyan ball, Cs atom; black ball, B atom; red ball, O atom; orange/olive tetrahedron, corner-/edge-sharing [BO_4_]; purple tringle [BO_3_].

CsB_5_O_8_. CsB_5_O_8_ is another alkali metal borate prepared under high-pressure (6 Gpa) and high-temperature (1173 K) conditions in a Walker-type multianvil apparatus [81]. Structurally, CsB_5_O_8_ features a similar structure to the aforementioned AB_3_O_5_ series. The basic B–O building blocks of CsB_5_O_8_ are corner-sharing [BO_3_], corner-sharing [BO_4_], and edge-sharing [BO_4_]; these units exhibit a ratio of 2:1:2, respectively (Figure 9a). The structure of CsB_5_O_8_ exhibits a 3D B–O covalent framework with Cs ions locating in the structural channels (Figure 9b).

**Figure 10:**
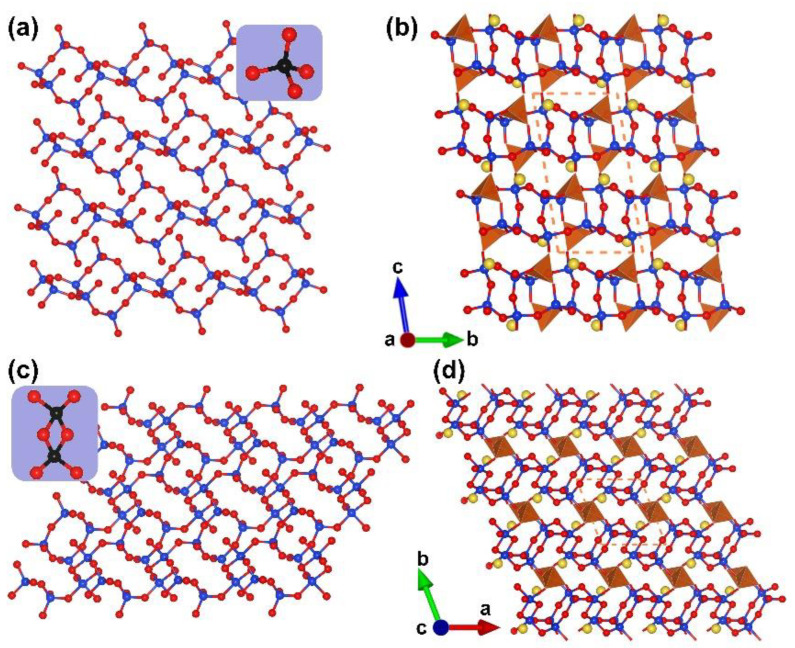
(**a**) The ^2^[Si_3_O_8_]_∞_ pseudo layer and [BO_4_] linkage in the structure of 16.2 Gpa-NaBSi_3_O_8_; (**b**) the view of the whole structure of 16.2 Gpa-NaBSi_3_O_8_ along [100] direction; (**c**) the ^2^[Si_3_O_8_]_∞_ layer and [B_2_O_6_] linkage in the structure of 24.8 Gpa-NaBSi_3_O_8_; (**d**) the view of the whole structure of 24.8 Gpa-NaBSi_3_O_8_ along [001] direction. Key: yellow ball, Na atom; blue ball, Si atom; black ball, B atom; red ball, O atom; orange tetrahedron, edge-sharing [BO_4_].

NaBSi_3_O_8_. In 2022, Gorelova et al. studied the high-pressure modification of NaBSi_3_O_8_, and revealed the transformation behaviors of NaBSi_3_O_8_ during continuous pressure increase [82]. Unexpectedly, above 27.8 GPa the crystal structure of NaBSi_3_O_8_ achieves the coexistence of the rare edge-sharing [BO_4_] tetrahedra and earlier unknown edge-sharing [SiO_5_] square pyramids. The structures under 16.2 and 27.8 Gpa are quite different. Both the 16.2 Gpa- and 27.8 Gpa-phases crystallize in the *P*$\bar{1}$. The Si atoms in the 16.2 Gpa-phase are all four-coordinated, and the corner-sharing [SiO_4_] tetrahedra are incorporated into the 1D [Si_3_O_8_]_∞_ chains, while 1/3 Si atoms are five-coordinated in the 27.8 Gpa-phase. These [SiO_5_] square pyramids are dimerized into [Si_2_O_8_] units (Figure 10a,c). SiO_4_ tetrahedra undergo geometrical distortion leading to the formation of SiO_5_ polyhedra due to the pressure-induced transformations. The [BO_4_] tetrahedra in 16.2 Gpa-phase and the [B_2_O_6_] dimers in 27.8 Gpa-phase act as linkers and further stable the whole structures (Figure 10b,d).

**Figure 11:**
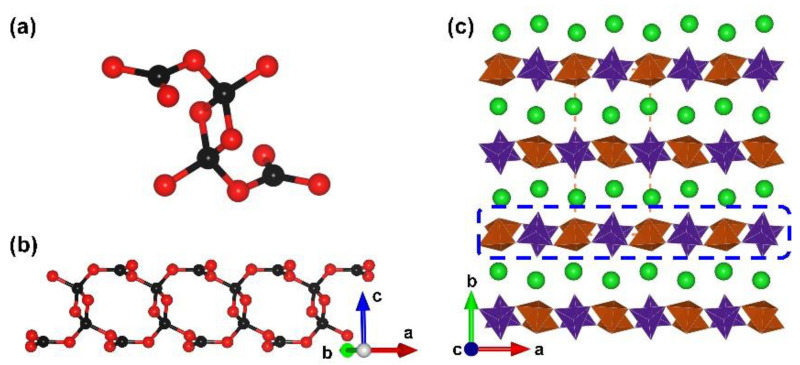
(**a**) The [B_4_O_10_] FBB of *γ*-BaB_2_O_4_; (**b**) the view of 1D ^1^[BO_2_]_∞_ chain in the structure; (**c**) the total structure of *γ*-BaB_2_O_4_ along [001] direction. Key: green ball, Ba atom; black ball, B atom; red ball, O atom; orange tetrahedron, edge-sharing [BO_4_]; purple tringle [BO_3_].

*γ*-BaB_2_O_4_. The *α*- and *β*-phases of barium metaborate are famously commercialized birefringent and nonlinear optical materials. Relevant theoretical studies offered various predicted phase of barium metaborate. In 2022, the third phase, *γ*-BaB_2_O_4_, was synthesized experimentally by Bekker et al. under conditions of 3 GPa and 1173 K [83]. *γ*-BaB_2_O_4_ crystallizes in a centrosymmetrical space group, *P*2_1_/*n* (no. 14). Its anionic B–O skeleton exhibits 1D chains, which is completely different from the isolated [B_3_O_6_] planar cluster in *α*- and *β*-phases. The [B_4_O_10_] FBB in *γ*-BaB_2_O_4_ is comprised of one [B_2_O_6_] ring and two additional [BO_3_] triangles (Figure 11a); these [B_4_O_10_] FBBs further assemble into the ^1^[B_2_O_4_]_∞_ chains (Figure 11b). Finally, the [BaO_10_] polyhedra stable the ^1^[B_2_O_4_]_∞_ chains in the lattice to form the total 3D structure of *γ*-BaB_2_O_4_ (Figure 11c). The calculated band gap is up to 7.045 eV, which implies transparency in the deep-UV region. The most intense band at a frequency of 853 cm^−1^ in the Raman spectra corresponds to the symmetric bending mode of the B−O−B−O ring in edge-sharing tetrahedra.

**Figure 12:**
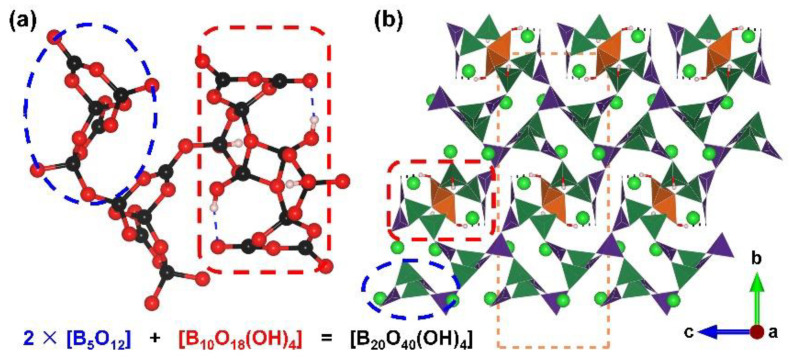
(**a**) The [B_20_O_40_(OH)_4_] FBB comprised with two [B_5_O_12_] clusters and one [B_10_O_18_(OH)_4_] cluster; (**b**) the total structure of *α*-Ba_3_[B_10_O_17_(OH)_2_] along [100] direction. Key: green ball, Ba atom; black ball, B atom; red ball, O atom; small pink ball, H atom; orange/olive tetrahedron, edge/vertex-sharing [BO_4_]; purple triangle [BO_3_].

*α*-Ba_3_[B_10_O_17_(OH)_2_]. Apart from the extreme high pressure afforded by the multi-anvil high-pressure device, the hydrothermal reactor can also provide relatively high pressure. In 2019, Lii et al. reported the structures of *α*-Ba_3_[B_10_O_17_(OH)_2_], which were obtained through hydrothermal reactions at 773 K and 0.1 Gpa. *α*-Ba_3_[B_10_O_17_(OH)_2_]. The phase containing edge-sharing [BO_4_] tetrahedra crystallizes in the monoclinic space group, *P*2_1_/*n* (no. 14), and presents a hydrated 3D B–O framework with Ba ions filling in the cavities (Figure 12b) [84]. In terms of its FBB, the complex [B_20_O_40_(OH)_4_] can be divided into the double [B_5_O_12_] (the blue dotted ellipse part) and [B_10_O_18_(OH)_4_] (the red dotted blanket) categories (Figure 12a). Unlike FBBs mentioned in other borates, the [B_2_O_4_(OH)_2_] units act as two connected nodes in the structure as the targeted [B_2_O_6_] units are bounded to hydrogen atoms as terminal hydroxy groups. The aggregation of [B_5_O_12_] clusters expanding in the *ac* plane leads to a corrugated layer, and the hydrated [B_10_O_18_(OH)_4_] clusters connect the adjacent antiparallel layers to form the ^3^[B_10_O_17_(OH)_2_]_∞_ covalent skeleton.

## 3. Ambient Pressure Synthesis of Borates with Edge-Sharing [BO_4_] Tetrahedra

The edge-sharing [BO_4_] tetrahedra-containing borates obtained from classical high-temperature solution and cooling method make it possible to obtain this species more conveniently. More importantly, borates obtained under ambient pressure might incorporate more *π*-conjugated [BO_3_] units. Edge-sharing borates with high [BO_3_]:[BO_4_] ratios, such as *β*-CsB_9_O_14_ (7:2) and Ba_6_Zn_6_(B_3_O_6_)_6_(B_6_O_12_) (22:2), are identified as birefringent crystals.

**Figure 13:**
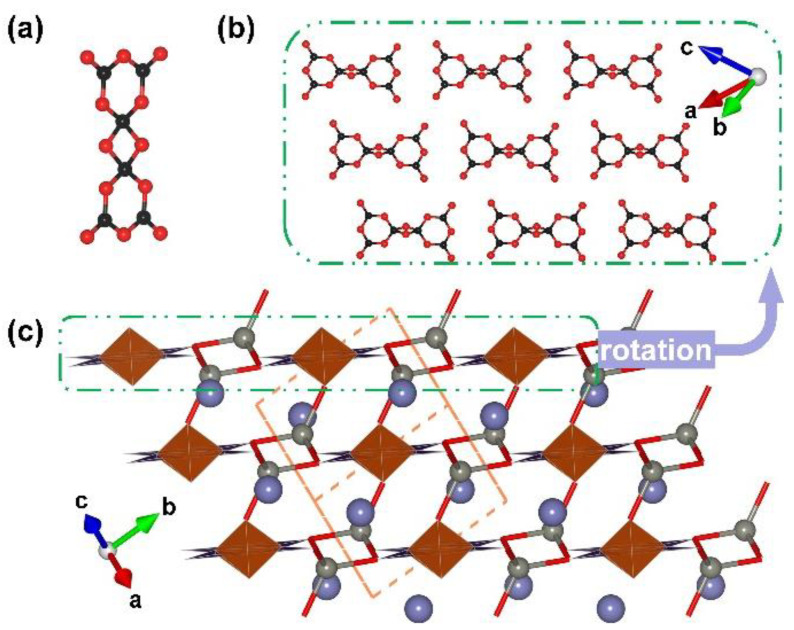
(**a**) The [B_6_O_12_] FBB; (**b**) the 2D [B_6_O_12_]_∞_ pseudo layer; (**c**) the total structure of KZnB_3_O_6_ along [110] direction. Key: purple ball, K atom; grey ball, Zn atom; black ball, B atom; red ball, O atom; orange tetrahedron, edge-sharing [BO_4_]; purple tringle [BO_3_].

KZnB_3_O_6_. The first case of borate containing edge-sharing [BO_4_] tetrahedra was synthesized under atmospheric pressure. KZnB_3_O_6_ was reported by Chen et al. and Wu et al. independently in 2010 [51,85]. KZnB_3_O_6_ crystallizes in the triclinic space group (*P*$\bar{1}$, no. 2) with a low symmetry. The [B_6_O_12_] FBB features isolated B–O cluster containing four [BO_3_] tringles and two edge-sharing [BO_4_] tetrahedra (Figure 13a). The aligned repetition of isolated [B_6_O_12_] clusters in the lattice gives a 2D [B_6_O_12_]_∞_ pseudo layer (see the green dotted blankets in Figure 13b,c), the pairs of edge-sharing [ZnO_4_] polyhedra connect the adjacent six [B_6_O_12_] clusters to form the ^3^[ZnB_3_O_6_]_∞_ network with K cations filling the cavities. Later, KZnB_3_O_6_ was defined as highly thermally stable by Chen et al., and its unidirectional thermal expansion property was investigated. Its unique property is explained by the cooperative rotations of rigid groups [B_6_O_12_] and [Zn_2_O_6_] driven by anharmonic thermal vibrations of K ions [86,87,88]. The discovery of KZnB_3_O_6_ indicated that high pressure is not essential for obtaining edge-sharing [BO_4_] tetrahedra-containing borates, and subsequently, ambient pressure edge-sharing [BO_4_] tetrahedra-containing borates have been synthesized successfully one after another.

**Figure 14:**
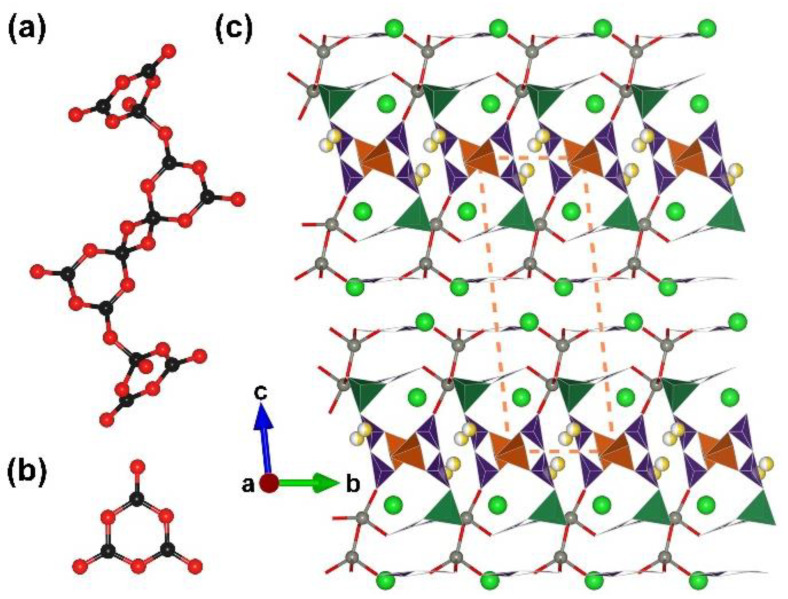
Two types of different FBBs occur in Ba_4_Na_2_Zn_4_(B_3_O_6_)_2_(B_12_O_24_): (**a**) [B_12_O_24_] FBB; (**b**) [B_3_O_6_] FBB; (**c**) the complex layered structure of Ba_4_Na_2_Zn_4_(B_3_O_6_)_2_(B_12_O_24_) along [100] direction. Key: green ball, Ba atom; yellow ball, Na atom; grey ball, Zn atom; black ball, B atom; red ball, O atom; olive/orange tetrahedron, corner-/edge-sharing [BO_4_]; purple tringle [BO_3_].

Ba_4_Na_2_Zn_4_(B_3_O_6_)_2_(B_12_O_24_). Ba_4_Na_2_Zn_4_(B_3_O_6_)_2_(B_12_O_24_) is another edge-sharing [BO_4_] tetrahedra-containing borate obtained without an extreme pressure condition, as reported by Chen et al. in 2013 [89]. Ba_4_Na_2_Zn_4_(B_3_O_6_)_2_(B_12_O_24_) crystallizes in the triclinic space group, *P*$\bar{1}$ (no. 2); it features a complex sandwich-like layered structure. There are two kinds of FBBs in the structure of Ba_4_Na_2_Zn_4_(B_3_O_6_)_2_(B_12_O_24_), namely [B_12_O_24_] and [B_3_O_6_], respectively (Figure 14a,b). The aggregation of [B_12_O_24_] FBBs and [Zn(1)O_4_] tetrahedra according to the stoichiometric ratio of 1:2 gives a layered [Zn_2_(B_12_O_24_)]_∞_ configuration expanding in the *ab* planes, while the second FBBs [B_3_O_6_] are connected to [Zn(2)O_4_] to form [Zn(B_3_O_6_)]_∞_ sheets. The assembly of two [Zn(B_3_O_6_)]_∞_ sheets and one [Zn(B_12_O_24_)]_∞_ layer leads to the formation of a complex [Zn_4_(B_3_O_6_)_2_(B_12_O_24_)]_∞_ sandwiched structure. Split Na(1,2) atoms with the occupancy of 0.47:0.53 fill in the cavities of the sandwiched layers, and spherical coordinated Ba ions fill in the adjacent sandwiched layers (Figure 14c).

**Figure 15:**
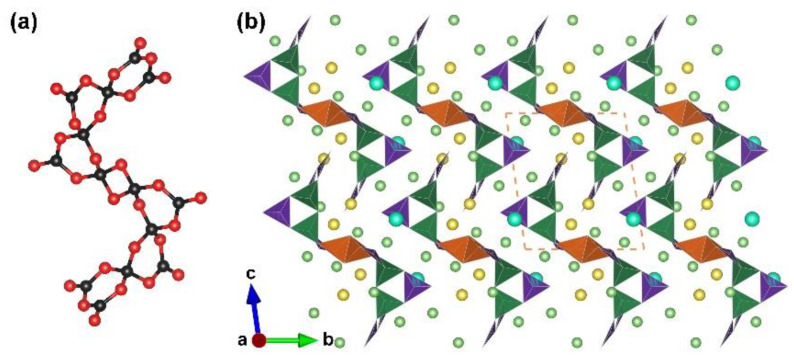
(**a**) The [B_14_O_28_] FBB; (**b**) view of the whole crystal structure of Li_4_Na_2_CsB_7_O_14_ along [100] direction. Key: green ball, Li atom; yellow ball, Na atom; cyan ball, Cs atom; black ball, B atom; red ball, O atom; orange/olive tetrahedron, edge-/corner-sharing [BO_4_]; purple tringle [BO_3_].

Li_4_Na_2_CsB_7_O_20_. The trimetallic borate Li_4_Na_2_CsB_7_O_20_ was reported by Pan et al. in 2019, and its expansion rate was investigated at the same time [90]. Li_4_Na_2_CsB_7_O_20_ crystallizes in a triclinic crystal system with the space group of *P*$\bar{1}$ (no. 2). With respect to its unique [B_14_O_28_] FBB, the centered [B_2_O_6_] ring acts as a four-connected node and further connects with one [BO_3_] tringle and one [B_5_O_11_] cluster (Figure 15a). The total crystal structure of Li_4_Na_2_CsB_7_O_20_ displays a 3D configuration with monovalent alkali metal Li, Na, and Cs ions residing in the free spaces (Figure 15b). The temperature-dependent unit cell parameters were collected experimentally. as Additionally, the theoretical evaluation of thermal expansion along the principal axes indicate the highly anisotropic thermal expansion behavior of Li_4_Na_2_CsB_7_O_20_. The expansion rates for *X*_1_, *X*_2_, and *X*_3_ were evaluated to be 3.51 × 10^−6^, 17 × 10^−6^, and 25.4 × 10^−6^ K^−1^, respectively. This compound may be used as a thermal expansion material.

**Figure 16:**
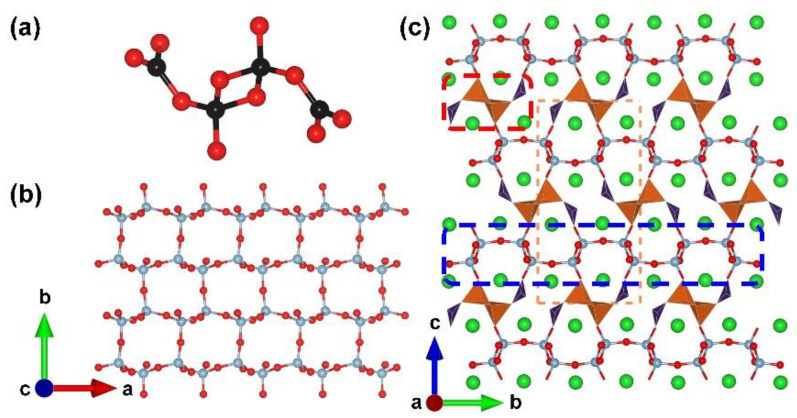
(**a**) The [B_4_O_10_] FBB of BaAlBO_4_; (**b**) the 2D [Al_2_O_5_]_∞_ layer constructed by vertex-sharing [AlO_4_] tetrahedra expanding in the ab plane; (**c**) the total structure of BaAlBO_4_ along [100] direction. Key: green ball, Ba atom; light blue ball, Al atom; black ball, B atom; red ball, O atom; small pink ball, H atom; orange tetrahedron, edge-sharing [BO_4_]; purple tringle [BO_3_].

BaAlBO_4_. In 2019, Pan et al. reported the synthesis and experimental and theoretical studies of an edge-sharing [BO_4_] tetrahedra-containing aluminum oxyborate, BaAlBO_4_. BaAlBO_4_ was synthesized via the high-temperature solution method under atmospheric pressure [91]. Single-crystal X-ray diffraction analysis reveals that BaAlBO_4_ crystallizes in a monoclinic space group, *P*2_1_/*c* (no. 14). The crystal structure of BaAlBO_4_ exhibits a 3D framework, which is comprised with [AlO_4_] tetrahedra, [B_4_O_10_] clusters, and A-site Ba^2+^ cations filling the structural channels. The corner-sharing [AlO_4_] units in the *ab* plane give a 2D ^2^[Al_2_O_5_]_∞_ layer with six MRs (Figure 16b). The [B_2_O_6_] rings connect with two [BO_3_] tringles end to end to form the isolated [B_4_O_10_] cluster (Figure 16a), which can be considered as the FBB of BaAlBO_4_. The combination of [B_4_O_10_] clusters and the neighboring ^2^[Al_2_O_5_]_∞_ layer give the final 3D framework.

**Figure 17:**
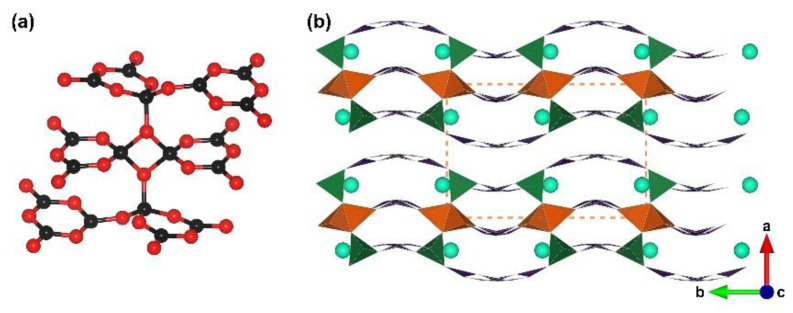
(**a**) The [B_18_O_34_] FBB of *β*-CsB_9_O_14_; (**b**) view of the whole crystal structure of *β*-CsB_9_O_14_ along [100] direction. Key: cyan ball, Cs atom; black ball, B atom; red ball, O atom; orange/green tetrahedron, edge/corner-sharing [BO_4_]; purple tringle [BO_3_].

*β*-CsB_9_O_14_. In 2019, Pan et al. prepared the *β*-CsB_9_O_14_ under ambient pressure. This compound is the first case of triple-layered borate with edge-sharing [BO_4_] tetrahedra [92]. Taking the [B_6_O_12_] cluster in the KZnB_3_O_6_ as the prototype, the sandwich-like [B_18_O_34_] FBB can be evolved from the combination of one [B_6_O_12_] and two anti-parallel [B_6_O_12_] double-ring units (Figure 17a). The further aggregation of [B_18_O_34_] FBBs in the *bc* plane leads to the formation of corrugated layers with A-site Ba^2+^ cations residing in the channels; the whole structure of *β*-CsB_9_O_14_ is formed by stacking of these triple-layered sheets along [100] direction (Figure 17b). The B–O anionic skeleton of *β*-CsB_9_O_14_ possesses a high [BO_3_]:[BO_4_] (7:2) ratio; the layered structure as well as the well-aligned [BO_3_] units in the lattice lead to a large optical anisotropy. The experimental and theoretical studies indicate that *β*-CsB_9_O_14_ can be identified as a potential deep-ultraviolet birefringent material with a wide band gap (>6.72 eV) and large birefringence (0.115 or 0.135 at 1064 nm).

**Figure 18:**
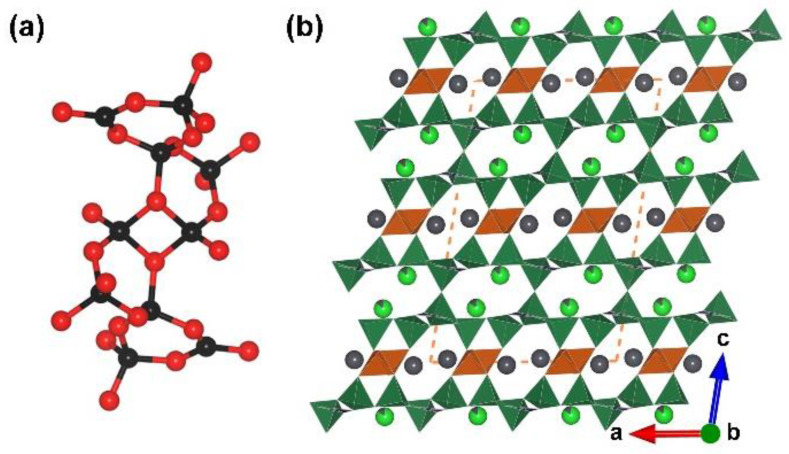
(**a**) The [B_10_O_24_] FBB of Pb_2.28_Ba_1.72_B_10_O_19_; (**b**) the view of the whole structure of Pb_2.28_Ba_1.72_B_10_O_19_ along [010] direction. Key: grey ball, Pb atom; green ball, Ba atom; black ball, B atom; red ball, O atom; orange/olive tetrahedron, edge/vertex-sharing [BO_4_].

Pb_2.28_Ba_1.72_B_10_O_19_. In 2021, an edge-sharing [BO_4_]-containing borate, Pb_2.28_Ba_1.72_B_10_O_19_, was obtained under ambient pressure by Pan et al. [93]. It features a 3D B–O anionic framework. Pb_2.28_Ba_1.72_B_10_O_19_ crystallizes in a monoclinic crystal system with the space group of *C*2/*c* (no. 15). Its asymmetric unit consists of one Pb atom, five B atoms, ten O atoms, and one common site of the Ba/Pb atom with the occupancy of 0.14:0.86. Unlike the [B_2_O_6_] basic ring in most of edge-sharing [BO_4_]-containing borate with four exocyclic O atoms acting as connection nodes, the centered [B_2_O_6_] in [B_10_O_24_] FBB connects two [BO_4_] tetrahedra by the two exocyclic *μ*_2_-O atoms and two [B_3_O_8_] by two endocyclic *μ*_3_-O atoms (Figure 18a). The whole [B_10_O_19_] anionic framework is assembled from [B_10_O_24_] FBBs and Pb and Ba ions located in the structural channels (Figure 18b).

**Figure 19:**
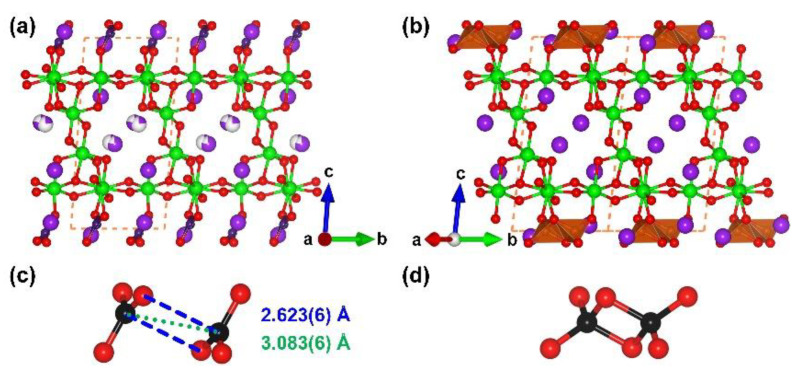
(**a**) The view of the whole structure of *β*-K_3_Sb_4_BO_13_ along [100] direction; (**b**) the view of the whole structure of *α*-K_3_Sb_4_BO_13_ along [100] direction; (**c**) the anti-parallel [BO_3_] pair in the *β*-K_3_Sb_4_BO_13_; (**d**) the edge-sharing [BO_4_] tetrahedra in the *α*-K_3_Sb_4_BO_13_. Key: purple ball, K atom; green ball, Sb atom; black ball, B atom; red ball, O atom; orange tetrahedron, edge-sharing [BO_4_]; purple triangle, [BO_3_].

K_3_Sb_4_BO_13_. In 2021, Quarez et al. discovered the complete transformation of adjacent [BO_3_] pairs into [B_2_O_6_] dimers in the *α*- and *β*-phase of K_3_Sb_4_BO_13_ driven by cooling [94]. The [BO_3_]-containing *α*-phase of K_3_Sb_4_BO_13_ is obtained from the traditional high-temperature solution method, and the symmetry increasing from *P*$\bar{1}$ to *C*2/*c* during the cooling process, accompanied with the transformation of two close [BO_3_] tringles into edge-sharing [B_2_O_6_] units. The structures of *α*- and *β*-K_3_Sb_4_BO_13_ display complex ^2^[Sb_4_O_13_]_∞_ layers separated by [BO_3_] pairs or edge-sharing [BO_4_] tetrahedra (Figure 19a,b). The anti-parallel [BO_3_] pair in the *α*-phase displays a short B⋯B distance (3.083(6) Å) and an extremely long B⋯O secondary bond (2.623(6) Å), while the coordination spheres of corresponding B atoms in the *β*-phase are distorted into tetrahedra (Figure 19c,d). The low temperature brings a lattice compression, which finally leads to B_2_O_6_ units, which shortens the B⋯B and B⋯O distances in each pair of adjacent BO_3_ triangles units. Further studies show that B K-edge electron energy loss (EELS) spectroscopes provide a characteristic signal of the B_2_O_6_ units; the EELS method may widely use to identify edge-sharing B_2_O_6_ units more convenient in the future.

**Figure 20:**
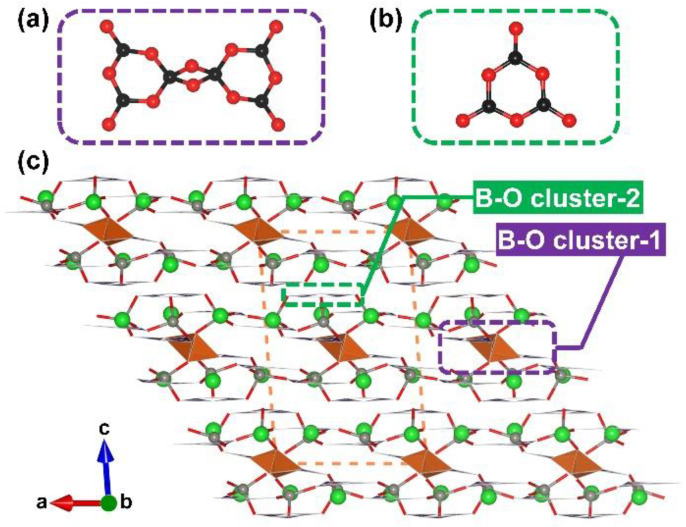
Two types of different FBBs occur in Ba_6_Zn_6_(B_3_O_6_)_6_(B_6_O_12_): (**a**) [B_6_O_12_] FBB; (**b**) [B_3_O_6_] FBB; (**c**) the structure of Ba_6_Zn_6_(B_3_O_6_)_6_(B_6_O_12_) along [010] direction. Key: green ball, Ba atom; grey ball, Zn atom; black ball, B atom; red ball, O atom; orange/green tetrahedron, edge-/corner-sharing [BO_4_]; purple tringle [BO_3_].

Ba_6_Zn_6_(B_3_O_6_)_6_(B_6_O_12_). Ba_6_Zn_6_(B_3_O_6_)_6_(B_6_O_12_) was reported by Mao et al. and Pan et al. independently in 2022 and identified as a potential birefringent crystal with a deep-ultraviolet absorption cut-off edge and strong optical anisotropy [95,96]. The structure of Ba_6_Zn_6_(B_3_O_6_)_6_(B_6_O_12_) features a 2D [ZnB_4_O_8_]_∞_ network constructed by [ZnO_4_] tetrahedra and two kinds of B–O clusters ([B_6_O_12_] and [B_3_O_6_]) with Ba cations located in the cavities (Figure 20). It should be noted that Ba_6_Zn_6_(B_3_O_6_)_6_(B_6_O_12_) shows extremely low symmetry (space group *P*-1, no. 2), and its asymmetric unit includes six Ba atoms, six Zn atoms, six planar [B_3_O_6_] clusters, and two [B_3_O_6_] fragments (half of [B_6_O_12_] cluster). To simplify the description of structure, we use B–O cluster-1 and B–O cluster-2 to represent the basic structural units (Figure 20a–c). In the sandwiched [ZnB_4_O_8_]_∞_ layers, the top and bottom of well-aligned [B_6_O_12_] clusters are shielded by the anti-parallel ^2^[Zn(B_3_O_6_)]_∞_ sheets. The …A-A’-A… stacking sequence of [ZnB_4_O_8_]_∞_ along the [001] direction leads to the formation of the total covalent skeleton, and Ba ions act as counterions in the lattice. From the structural perspective, the uniformly arrangement of two kinds of B–O clusters and the high ratio of highly birefringence-active [BO_3_] tringles and [BO_4_] tetrahedra (22:20) indicate that Ba_6_Zn_6_(B_3_O_6_)_6_(B_6_O_12_) may have remarkable optical anisotropy. In addition, the dangling bonds of terminal in two kinds of B–O clusters are eliminated by the covalent [ZnO_4_] tetrahedra; thus, the short-wavelength absorption cut off edge has a blue shift. The basic physical properties of Ba_6_Zn_6_(B_3_O_6_)_6_(B_6_O_12_) were also studied. The transmission/absorption spectra indicate that Ba_6_Zn_6_(B_3_O_6_)_6_(B_6_O_12_) possesses a wide transparency window from 180 nm to 3405 nm. The difference of refractive indices based on a (001) wafer at 589.3 nm is as large as 0.14, which indicates that the birefringence of Ba_6_Zn_6_(B_3_O_6_)_6_(B_6_O_12_) is even larger than the commercialized *α*-BaB_2_O_4_. Moreover, thermal analysis demonstrates that Ba_6_Zn_6_(B_3_O_6_)_6_(B_6_O_12_) melts congruently. The acquirement of bulk crystals could be anticipated as is evidenced by the already grown sub-centimeter sized crystals.

## 4. Conclusions

The synthesis of edge-sharing borates greatly changes the rule of corner sharing B–O units in borate structures, and further work demonstrates that the extreme synthetic conditions, such as high pressure, are not necessary for edge-sharing borates. The crystalline borates with edge-sharing [BO_4_] tetrahedra continue to develop; about 34 new edge-sharing borates containing edge-sharing B_2_O_6_ unit have been found in recent years, among which three are crystallized in noncentrosymmetric space groups, only about 10% in the whole edge-sharing borates. This ratio is much smaller than 35% for the entire borate system, which may be attributable to the [BO_4_] units likely formed under the high-pressure environment [97]. Noncentrosymmetric edge-sharing borates are needed to better understand the NLO property in these types of structures. Fortunately, more *π*-conjugated [BO_3_] units are found under the ambient-pressure environment; the high [BO_3_] and [BO_4_] ratio in edge-sharing borates may be beneficial for the formation of noncentrosymmetric structures.

The signal of the B_2_O_6_ structural motif can be unambiguously assigned in the B K-edge EELS spectrum. Some of these edge-sharing borates exhibit interesting properties, such as unusual anisotropic thermal expansion behavior. It is curious to chemists whether edge-sharing BO_3_/BO_4_, BO_3_/BO_3_, or even face-sharing B–O units can be realized in the future. It is also expected that the synthesis of edge-sharing [BO_3_F]^4−^, [BO_2_F_2_]^3−^, and [BOF_3_]^2−^ units in the future will greatly enrich the structural chemistry of crystalline fluorooxoborates. Finally, we should better understand the structure–property relationships of these edge-sharing borates, which will help us to find more applications.

## Data Availability

Not applicable.
